# Development and Application of a High-Throughput Method for the Purification and Analysis of Surface Carbohydrates from *Klebsiella pneumoniae*

**DOI:** 10.3390/biology13040256

**Published:** 2024-04-12

**Authors:** Francesca Nonne, Mariagrazia Molfetta, Rebecca Nappini, Chiara La Guidara, Roberta Di Benedetto, Siwaphiwe Mfana, Barbara Bellich, Maria Michelina Raso, Gianmarco Gasperini, Renzo Alfini, Paola Cescutti, Francesco Berlanda Scorza, Neil Ravenscroft, Francesca Micoli, Carlo Giannelli

**Affiliations:** 1 GSK Vaccines Institute for Global Health, 53100 Siena, Italy; mariagrazia.x.molfetta@gsk.com (M.M.); rebecca.x.nappini@gsk.com (R.N.); chiara.laguidara@unifi.it (C.L.G.); roberta.x.di-benedetto@gsk.com (R.D.B.); maria-michelina.m.raso@gsk.com (M.M.R.); renzo.x.alfini@gsk.com (R.A.); francesco.x.berlandascorza@gsk.com (F.B.S.); francesca.x.micoli@gsk.com (F.M.); carlo.x.giannelli@gsk.com (C.G.); 2 Department of Life Science, University of Trieste, 34127 Trieste, Italy; pcescutti@units.it; 3 Department of Chemistry, University of Cape Town, Cape Town 7700, South Africa; mfnsiw001@myuct.ac.za (S.M.); neil.ravenscroft@uct.ac.za (N.R.); 4 Department of Advanced Translational Diagnostics, Institute for Maternal and Child Health, IRCCS “Burlo Garofolo”, 34137 Trieste, Italy; barbara.bellich@burlo.trieste.it; 5 GSK, 53100 Siena, Italy; gianmarco.x.gasperini@gsk.com

**Keywords:** *Klebsiella pneumoniae*, O-antigen, K-antigen, polysaccharide purification

## Abstract

**Simple Summary:**

The work reported here focuses on addressing the need to develop a purification method of Kp polysaccharide antigens that can be applied universally to all serotypes regardless of their structure. This refined approach has demonstrated efficacy in level of purity without compromising structural integrity or labile substituents. This scalable method could help the development of polysaccharide-based vaccines against Kp.

**Abstract:**

*Klebsiella pneumoniae* (Kp) is a Gram-negative bacterium, and a leading cause of neonatal sepsis in low- and middle-income countries, often associated with anti-microbial resistance. Two types of polysaccharides are expressed on the Kp cell surface and have been proposed as key antigens for vaccine design: capsular polysaccharides (known as K-antigens, K-Ags) and O-antigens (O-Ags). Historically, Kp has been classified using capsule serotyping and although 186 distinct genotypes have been predicted so far based on sequence analysis, many structures are still unknown. In contrast, only 11 distinct OAg serotypes have been described. The characterization of emerging strains requires the development of a high-throughput purification method to obtain sufficient K- and O-Ag material to characterize the large collection of serotypes and gain insight on structural features and potential cross-reactivity that could allow vaccine simplification. Here, this was achieved by adapting our established method for the simple purification of O-Ags, using mild acetic acid hydrolysis performed directly on bacterial cells, followed by filtration and precipitation steps. The method was successfully applied to purify the surface carbohydrates from different Kp strains, thereby demonstrating the robustness and general applicability of the purification method developed. Further, antigen characterization showed that the purification method had no impact on the structural integrity of the polysaccharides and preserved labile substituents such as O-acetyl and pyruvyl groups. This method can be further optimized for scaling up and manufacturing to support the development of high-valency saccharide-based vaccines against Kp.

## 1. Introduction

*Klebsiella pneumoniae* (Kp) is a Gram-negative bacterium of the *Enterobacteriaceae* family, that causes an array of human diseases including pneumonia, urinary tract infection, and bacteremia in neonates and immunocompromised hosts, particularly in healthcare settings [1,2]. Kp represents a serious public health problem worldwide and has recently been recognized as the most common pathogen contributing to infectious deaths in young children, largely by causing neonatal sepsis [3,4]. The World Health Organization (WHO) has ranked *K. pneumoniae* as a “critical-priority 1” antibiotic-resistant pathogen, and it is also classified as one of the “ESKAPE” pathogens [5,6]. Infection treatments are increasingly limited by the high prevalence of antibiotic resistance, and to date, there are no licensed vaccines against Kp. Many of the vaccine candidates designed over the years have targeted either capsular polysaccharides or O-antigens (O-Ags), the two polysaccharide antigens exposed on Kp cell surfaces [7], representing important virulence factors involved in the immune evasion mechanism of Kp. The capsule type, also known as the K-antigen (K-Ag) or K-type, is historically used to indicate the Kp serotype. However, due to the great diversity of Kp serotypes, with geographical and longitudinal potential variability, there is a need for a high-valent vaccine with >20 K-Ag and/or at least 4 OAg to provide enough coverage [7]. This poses challenges to the development of an affordable vaccine, especially for LMICs, and requires the use of generic methodologies easily applicable to polysaccharides with different structures.

Indeed, to date, 186 distinct K-Ags have been identified based on their K-locus (KL) sequence, and only 79 of these have been characterized and classified [8,9]. Chemical structure studies reveal that most Kp K-Ag repeating units (RUs) have a length ranging from three to six monosaccharides [10]. One of the sugars is usually represented by an acid component, such as glucuronic acid (GlcA) or galacturonic acid (GalA), which makes the Kp capsule anionic. The other monosaccharides consist of neutral sugars such as galactose, glucose, mannose, rhamnose, and occasionally fucose. Polysaccharide decorations, consisting mainly of a pyruvate moiety but also O-acetyl, O-lactyl, O-formyl, and glutamate groups, contribute to the variety and antigenic diversity of Kp capsules [10,11]. The role of pyruvate in immunogenicity has been scarcely explored; however, it is reported to be crucial for bacteria metabolism, virulence, and growth [12]. Conversely, despite the fact that it cannot be generalized for all microorganisms, O-acetylation was highlighted as extremely important for the immunogenicity and pathogenicity of different bacteria [13]. The role of such structural modifications for Kp K-Ag needs to be further investigated, although pyruvylation and O-acetylation were indicated as essential for cytokine induction [14]. Moreover, O-acetylation seems to contribute to Kp pathogenicity by promoting the cell adhesion of bacteria [15].

Unlike the wide variety of K-Ags, only 11 O-Ags have been identified with a limited range of structures: O1v1, O1v2, O2v1 (or 2a), O2v2 (or O2afg), O3, O4, O5, O7, O8, O9 (or O2aeh), and O12 [16,17,18]. O1 and O2 are described as variant 1 (v1) and variant 2 (v2). O1v1 contains D-galactan I [ → 3)-β-D-Gal*f*-(1 → 3)-α-D-Gal*p*-(1 →] and D-galactan II [→ 3)-α-D-Gal*p*-(1 → 3)-β-D-Gal*p*-(1 →] disaccharide repeating units. O2v1 (or 2a) is made up of a shorter repeating unit composed only of D-galactan I. O1v2 and O2v2 are a branched version of their v1 and consist of the addition of galactose to D-galactan I, to build the [→ 3)-β-D-Gal*f*-(1 → 3)-[α-D-Gal*p*-(1 → 4)]-α-D-Gal*p*-(1 →] trisaccharide repeating unit, indicated as D-galactan III [19]. Conversely, O3 and O5 O-Ags are composed of mannose homopolymers. O3 is further divided into serotypes O3, O3a, and O3b, differing in the number of mannose residues and consisting, respectively, of a penta-, tetra-, and tri-saccharide RU based on the polymerization at the non-reducing end [20]. Similarly to O3b, the O5 antigen is made up of a tri-mannose chain but differs in anomeric configurations and glycosidic linkages. The non-reducing ends of both O3 and O5 RUs are terminated by methylphosphate (MeP) and methyl (Me) groups, respectively [21].

Polysaccharide purification is the first step for the development of a vaccine against *Klebsiella*. The identification of the most appropriate purification strategy usually depends on polysaccharide structures and properties. The aim of this work was to develop a simple purification process generally applicable to Kp polysaccharides (K-Ags and O-Ags) independently from their specific structural features, to obtain sufficient and highly pure material while ensuring the preservation of structural integrity. At this stage of development, the impact of decorations, e.g., O-acetyl and pyruvate groups, on immunogenicity is not clear, and it is fundamental to preserve the original polysaccharide structure during isolation and purification steps.

The method developed can not only support the faster development of a high-valent polysaccharide-based vaccine against Kp, but also allow the characterization of a large collection of clinical isolates to better understand Kp serotype variability, determine unknown structures, and assess the potential cross-reactivity among different serotypes that could allow for vaccine simplification.

## 2. Experimental Section

### 2.1. Chemicals

Sodium chloride (NaCl), sodium sulphate (Na_2_SO_4_), sodium phosphide (NaPi), and deuterium oxide (D_2_O) were from Sigma-Aldrich (St. Louis, MO, USA); calcium chloride (CaCl_2_) and cetyl trimethylammonium bromide (CTAB) were from Carlo Erba; ethanol (EtOH), acetic acid (AcOH), acetonitrile (ACN), 3 M Hydrochloric acid (HCl), Silylating mixture, methanol, Dimethyl sulfoxide (DMSO), pyridine, hexane, chloroform, iodomethane, and acetic anhydride were from Merck (Darmstadt, Germany); Sodium hydroxide (J. T. Baker, Avantor, Radnor, PA, USA), Micro BCA Reagent Kit (Thermo Fisher Scientific, Waltham, MA, USA), Trifluoroacetic acid (VWR, Radnor, PA, USA), sodium borodeuteride (ACROS Organics-Thermo Fisher Scientific, Waltham, MA, USA), and Potassium Hydride (Thermo Fisher Scientific, Waltham, MA, USA).

### 2.2. Culture Supernatants

#### 2.2.1. K-Ag Isolation

Kp strains acquired from Public Health England (NCTC11228, NCTC13810, NCTC9135, NCTC9145, NCTC9182, and NCTC8172) or kindly received from SSI (12641B, 17648, 27870B, 19679, 24745-2, 10405, 773, 30405, 34473, 4998, and 5765B) were plated on Worfel-Ferguson Agar plates (yeast extract 2 g/L, MgSO_4_⸱7H_2_O 0.25 g/L, K_2_SO_4_ 1 g/L, NaCl 2 g/L, and sucrose 20 g/L) [22] and incubated overnight (ON) at 30 °C. After growth, the bacterial biomass was collected with a solution of AcOH 1% (*v*/*v*) (about 3 mL per plate), and the suspension was incubated for 6 h at 100 °C in a preheated thermoblock heater. The material was then centrifuged (4000 rpm, 30 min, 4 °C), and the polysaccharide-containing supernatant was collected and filtered with 0.22 µm membranes. 

#### 2.2.2. O-Ag Isolation

Kp strains received from SSI (7008B, 1217, 24649, 25827, and 29867) were inoculated ON in LB medium (30 °C, 180 rpm) and then diluted at OD_600_ = 0.1 in M63C medium (composed of (NH_4_)_2_SO_4_ 2 g/L, KH_2_PO_4_ 13.6 g/L, FeSO_4_⸱7H_2_O 0.5 mg/L, 1 mM MgSO_4_, 0.0005% vitamin B1, 0.1% casamino acids, and 0.4% glycerol) and incubated for 24 h at 37 °C, 180 rpm [23]. Then, AcOH was added to the culture broth to a final concentration of 2% *v*/*v* and the material was incubated for 2 h at 100 °C in a preheated thermoblock heater [24]. The material was then centrifuged (14,000 rpm, 10 min, 4 °C) and the supernatant collected and filtered through 0.22 µm membranes. 

### 2.3. Polysaccharide Purification

#### 2.3.1. K-Ag Purification

The bacterial supernatant was dried by means of lyophilization and then resolubilized in water. The solution obtained was adjusted to a final concentration of 240 mM NaCl, 5 mM Na_2_SO_4_, CTAB 1%, using 1 M NaCl, 100mM Na_2_SO_4_, and 10% CTAB, and heated at 100 °C for 4 h. Once cooled at 4 °C for 10 min, the solution was centrifugated at room temperature (RT), 4000 rcf for 15 min, and the supernatant separated from the pellet, diluted 1:10 with 5 mM Na_2_SO_4_, and heated at 37 °C for 1 h. After centrifugation (15 min, 4000 rcf, RT), the pellet containing the K-Ag was solubilized in 1 M CaCl_2_. Pure EtOH 98% was added to the solution to a final concentration of 25% (*v*/*v*). After 30 min at RT, the supernatant was collected by centrifugation (15 min, 4000 rcf, RT) and diluted with EtOH 98% to a final concentration of 80% (*v*/*v*). After 1 h at RT, the pellet containing the K-Ag was isolated by centrifugation (15 min, 4000 rcf, RT) and solubilized in 1 M NaCl. Finally, the solution was further purified using an Amicon 10K ultra centrifugal filter unit (Millipore, Burlington, MA, USA) and further filtered with 0.22 µm Millipore filters (Millipore, Burlington, MA, USA).

#### 2.3.2. O-Ag Purification

The bacterial supernatant was purified by means of Tangential Flow Filtration (TFF), using a 10 K molecular weight cut-off (MWCO) Hydrosart (200 cm^2^) membrane cassettes from Sartorius (Göttingen, Germany). The material was concentrated to 40 mL (permeate flow: from 7.7 to 9.2 mL/min; Pin 1 bar; Pout 0.54 bar), followed by 20 volumes of diafiltration against 1 M NaCl buffer and 10 cycles of diafiltration against water (permeate flow: from 7.7 to 9.2 mL/min; Pin 1 bar; Pout 0.54 bar). Next, sodium acetate buffer 250 mM pH 3.7 was added to the solution to have a final acetate concentration of 2.5 mM. After mixing at RT for 30 min, the supernatant was collected by means of centrifugation and filtered using cationic exchange (CEX) chromatography with Sartobind S MA75 filters (Sartorius (Göttingen, Germany). After pH neutralization, an anion exchange chromatography with a HiTrap Q FF column (Cytiva, Chicago, IL, USA) was applied to the solution. A second purification using TFF was finally performed on the purified OAg solution for a buffer exchange, following the same condition reported above.

### 2.4. Analytical Methods

#### 2.4.1. Intermediate Characterization

The polysaccharide concentration of all purification intermediates was determined by means of HPAEC-PAD as described later, and it was used for the calculation of step yields. Protein concentration was measured by means of micro BCA, using bovine serum albumin (BSA) as a reference, following the manufacturer’s instructions. DNA content was estimated by measuring UV absorbance at a wavelength of 260 nm with a Lambda 25 spectrophotometer (Perkin Elmer, Waltham, MA, USA), assuming that a nucleic acid concentration of 50 mg/mL produces an OD_260_ = 1. This may overestimate nucleic acid content, in part due to other UV absorbing impurities; however, it is a useful and fast method to quantify nucleic acid impurities. The % of protein and nucleic acid impurities compared to total saccharide content was calculated. Supernatant separated from the bacterial pellet was lyophilized and resuspended in water to quantify the initial amount of PS. Proteins and nucleic acids were not calculated because the composition of the bacterial growth interfered with the analyses. After the first precipitation with CTAB, the intermediate was diluted with NaCl 240 mM in order to prevent PS precipitation in the analyses performed.

#### 2.4.2. High-Performance Liquid Chromatography–Size-Exclusion Chromatography (HPLC–SEC)

The molecular size distribution of all purified K- and O-Ags was measured using an HPLC-SEC TSK gel 6000PW (30 cm × 7.5 mm) column connected in series with a TSK gel 3000 PW_XL_ column (30 cm × 7.8 mm), and a TSK gel PW_XL_ guard column (4.0 cm × 6.0 mm) was used for K-Ag molecular weight estimation, while a TSK gel G3000 PW_XL_ column (30 cm × 7.8 mm) with a TSK gel PW_XL_ guard column (4.0 cm × 6.0 mm) was used for O-Ags. In both cases, pullulans were used to build standard calibration curves (2000–50 kDa range for K-Ags and 200–6 kDa for O-Ags) and 0.1 M NaCl, 0.1 M NaH_2_PO_4_, 5% ACN, pH 7.2 buffer was used as mobile phase at the flow rate of 1 mL/min and 0.5 mL/min for K-Ag or O-Ag runs, respectively. The mode of the molecular weight distribution was reported for both polysaccharides. Polysaccharide peaks were detected by means of the differential refractive index (dRI). UV detection at 260 and 280 nm was used to follow the reduction of nucleic acid and protein impurities during the purification process. Additionally, fluorescence emission was detected at ex280/em336 nm to check protein impurities. Columns and guard columns were from Tosoh Bioscience GmbH (Griesheim, Germany).

#### 2.4.3. High-Performance Anion-Exchange Chromatography with Pulsed Amperometric Detection (HPAEC-PAD)

All samples, diluted to a final volume of 450 μL, to have each sugar monomer in the range 0.5–10 μg/mL, were hydrolyzed in 2 M trifluoroacetic acid (TFA) by using a Microwave Reaction System (Multiwave PRO, Anton Paar, Gratz, Austria) maitaining 120 °C for 30 min by using an irradiation power of 700 WThe heating time to reach target temperature was set to 10 min for all runs, cooling after the experiment was performed down to 55 °C in 5 min. After the hydrolysis, the samples were dried using Speedvac Vacuum concentrator (Thermo Fisher Scientific, Waltham, MA, USA), reconstituted in 450 μL of water, and filtered using 0.45-μm 96-well plate filters for chromatographic analysis. HPAEC–PAD was performed with a Dionex ICS5000 (Thermo Fisher Scientific, Waltham, MA, USA) equipped with a CarboPac PA1 column (2 × 250 mm) Thermo Fisher Scientific (Waltham, MA, USA) coupled with a PA1 guard column (2 × 50 mm) Thermo Fisher Scientific (Waltham, MA, USA). The separation of the sugars was performed with a flow rate of 0.375 mL/min, eluting in 18 mM NaOH over 15 min. Next, a 17 min gradient from 0 to 300 mM of Sodium Acetate (AcONa) in 150 mM NaOH was eluted. Finally, the column was re-equilibrated with 18 mM NaOH for 20 min. The effluent was monitored using an electrochemical detector in the pulse amperometric mode with a gold working electrode and an Ag/AgCl reference electrode. The resulting chromatographic data were processed using Chromeleon software 7.2. (Thermo Fisher Scientific, Waltham, MA, USA) Calibration curves were built with a sugar monomer mixture (Fuc, Rha, Man, GlcNAc, Glc, Man, GalA, and GlcA), each in the range 0.5–10 μg/mL. The standard was treated in the same way as the samples.

### 2.5. Sugar Composition and Linkage Analysis Using GLC and GLC-MS

Composition analysis was carried out after the methanolysis of the polysaccharide with 3 M HCl in methanol at 85 °C for 16 h followed by the chemical derivatization of the sample to trimethylsilyl methyl glycosides using the reagent Sylilating mixture (Merck) [25]. To determine the position of the glycosidic linkages, the polysaccharide was permethylated following the protocol developed by Harris et al. [26], hydrolyzed with 2 M TFA for 1 h at 125 °C, reduced with sodium borodeuteride (NaBD_4_), and peracetylated with acetic anhydride [27], to give a mixture of partially methylated alditol acetates. 

The derivatized samples were analyzed by means of GLC, using an Agilent Technologies 6850 (Santa Clara, CA, USA) gas chromatograph equipped with a flame ionization detector, using He as the carrier gas and a Zebron ZB-5 MSi capillary column 30 m × 250 μm × 0.25 μm (Phenomenex—Torrance, CA, USA). The following temperature programs were used: for trimethylsilyl methyl glycosides, 1 min at 150 °C, 150–280 °C at 5 °C/min, and 2 min at 280 °C; for partially methylated alditol acetates, 1 min at 90 °C, 90–140 °C at 25 °C/min, 140–200 °C at 5° C/min, 200–280 °C at 10 °C/min, and 10 min at 280 °C. GLC-MS analyses were carried out on an Agilent Technologies 7890 A (Santa Clara, CA, USA) gas chromatograph coupled to an Agilent Technologies 5975C VL MSD (Santa Clara, CA, USA), using the same column and the temperature programs of the GLC analyses. The values of the integrated area of the partially methylated alditol acetates were corrected using the effective carbon response factors [28].

### 2.6. NMR Analysis

The molecular size of all purified K-Ags was reduced using a Sonics Vibra cell (Bio Class, Pistoia, Italy) instrument for 30 min at 130 W. After the confirmation of size reduction by means of HPLC-SEC, the sample was lyophilized before re-solubiliziation in deuterium oxide (D_2_O). Purified O1v2 was lyophilized without any further treatment and re-solubilized in D_2_O.

For O-acetylation measurement, ^1^H NMR spectra were measured at 400 MHz and 298 K with a Bruker AvanceIII 400 MHz NMR spectrometer (Bruker, Billerica, MA, USA); ^1^H values are reported in ppm; the solvent peak for D_2_O was calibrated at 4.79 ppm. The standard Topspin 3.2 software (Bruker, Billerica, MA, USA) was used for spectra interpretation.

For structural determination, polysaccharide samples (2–10 mg) were dissolved in deuterium oxide (D_2_O) and subjected to two cycles of D_2_O exchange before being transferred to a 5 mm tube. Spectra were recorded on a Bruker Avance III 600 MHz NMR spectrometer (Billerica, MA, USA) equipped with a BBO Prodigy cryoprobe and processed using standard Bruker software (Topspin 3.2). The probe temperature was set at 323 or 343 K. One-dimensional (^1^H and ^13^C) and two-dimensional COSY, TOCSY, NOESY, HSQC, and HMBC NMR experiments were performed. The 2D COSY and NOESY experiments were recorded with pre-saturation of HOD, whereas the TOCSY experiments were performed using DOSY to remove signals from low-molecular-weight components (ledbpgpml2s2d). The 2D TOCSY experiments were recorded using a mixing time of 160 or 180 ms and the 1D variants using 200 ms. The 2D NOESY experiments and the 1D variants were recorded using a mixing time of 300 ms. The HSQC experiment was optimized for J = 145 Hz (for directly attached ^1^H-^13^C correlations), and the HMBC experiments optimized for a coupling constant of 6 Hz (for long-range ^1^H-^13^C correlations). To improve sensitivity by performing many scans, the 2D experiments were recorded using non-uniform sampling: 40% for homonuclear and 20–30% for heteronuclear experiments. Chemical shifts are expressed in ppm using acetone as internal reference (2.225 ppm for ^1^H and 31.07 ppm for ^13^C). 

## 3. Results

We have previously developed a general method for O-Ag extraction and purification from Gram-negative bacteria based on the direct acetic acid hydrolysis of intact bacterial cultures, without the previous isolation and inactivation of bacteria [24,29]. The acidic treatment inactivates bacteria and cleaves the labile linkage between the 2-keto-3-deoxyoctonate (KDO) at the end of the lipopolysaccharide core region linked to lipid A, releasing the O-Ag chains in the supernatant. As a starting point, the same procedure was applied here to simultaneously release O-Ag and K-Ag into the supernatant after bacterial growth. Bacteria were boiled with 1% AcOH at 100 °C for 6 h. The bacterial supernatant, containing the K-Ag, O-Ag, and core detached from lipid A, DNA, and proteins, was separated from the bacterial pellet through centrifugation.

### 3.1. K-Ag Purification

K-Ag purification was based on a fractional precipitation with the cationic surfactant cetyltrimethylammonium bromide (CTAB) [30,31]. CTAB establishes ionic interactions with negatively charged species, complexing them and causing their precipitation. Both DNA and K-Ag have negatively charged groups, but the former has higher charge density due to the presence of one phosphate group for each sugar unit, while the latter presents a negative charge every repeating unit, generally composed of three to six sugars. By varying the sodium chloride (NaCl) concentration in solution, it was possible to separately precipitate DNA and K-Ag, avoiding co-precipitation. With a salt concentration of 240 mM [30,31], CTAB establishes ionic interactions with high-molecular-weight DNA, making it precipitate, while K-Ag remains in solution. This initial step was performed at 100 °C to induce protein denaturation and avoid the aggregate formation of K-Ag with fimbrial proteins, which are difficult to further purify without the use of chlorinated solvent extraction. Indeed, high-molecular-weight protein aggregates were found with final purified K-Ag when this initial step was performed at 37 °C, and Western Blot analysis on purified K-Ag evidenced the presence of MrKA type 3 fimbriae (Figure S1).

In the second step of the fractional precipitation, the salt concentration was reduced by diluting the solution 10-fold, allowing for the CTAB to form a complex with K-Ag which is hydrophobic and precipitates. The K-Ag-CTAB pellet was then solubilized in 1 M CaCl_2_ or 1 M NaCl to replace CTAB with Ca^2+^ or Na^+^. The CTAB precipitation steps were performed in the presence of 5 mM Na_2_SO_4_, to accelerate precipitation [31]. An additional reduction of impurities was achieved by two more precipitation steps involving ethanol (EtOH) at different percentages. The CaCl_2_/NaCl solution was first taken to 25% EtOH final concentration, allowing the further reduction of lower molecular-weight DNA. Next, it was diluted to 80% EtOH final concentration for the precipitation of purified K-Ag from residual CTAB and proteins. The last step consisted of diafiltration against water with the further removal of very low-molecular-weight proteins and DNA fragments that were formed during AcOH hydrolysis. 

**Figure 1:**
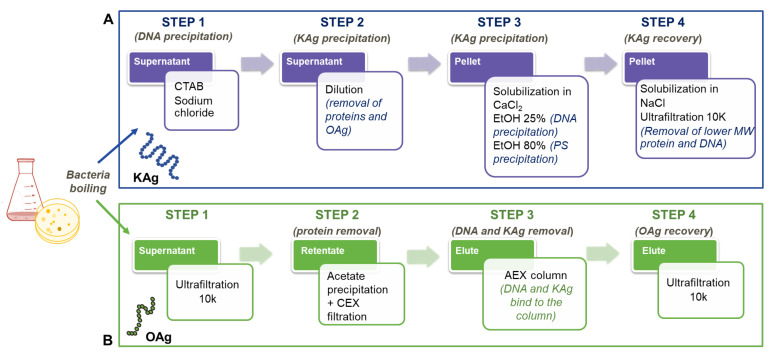
Schematic processes for K-Ag (**A**) and O-Ag (**B**) purification.

The purification process is shown in Figure 1A, while Table 1 reports the characterization of the intermediates from three different purifications performed on K2, K15, and K64 serotypes, confirming protein and DNA reduction step by step, according to what is described above. K-Ag recovery at each step is also indicated. Starting from the same batch of bacterial growth, the extraction of K64 was performed by boiling with and without acetic acid, and the intermediates were characterized, verifying that the process developed worked well even when the first step was performed in water only (Table 1).

K-antigens were extracted from K2, K62, and K64 serotypes by boiling both with and without acetic acid, to verify the impact of the acid treatment on PS size and structural integrity. In all cases, HPLC-SEC analysis indicated molecular size reduction using acetic acid and slightly higher yields (Figure S2 and Table 2). The treatment with acetic acid facilitated the separation of the bacterial pellet by means of centrifugation. For K2, the structural integrity was confirmed, and very similar O-acetylation levels were found both with and without acetic acid (Figure S3A and Table 2). For K64, the acetic acid treatment did not impact the level of O-acetylation but strongly affected the level of pyruvate decoration (Figure S3B). This could result in conformational changes of the PS chains, explaining the stronger impact on the apparent molecular size by HPLC-SEC observed for K64 with respect to K2 and K62 (Table 2). The developed procedure was further applied to the purification of an additional 11 serotypes, many of them with unknown structures, resulting in a high level of purity for all of them (Table 3).

### 3.2. OAg Purification

For OAg purification, the method previously developed was slightly modified to separate the OAg chains from negatively charged K-Ag chains. The supernatant was first purified by means of TFF (10K membrane cut-off) to remove low-molecular-weight mass impurities and the core population. Then, protein and nucleic acid impurities were reduced by means of precipitation with AcOH at pH 3. The supernatant was collected by means of centrifugation and filtered using cationic exchange (CEX) chromatography to remove residual proteins. After pH neutralization, an anion exchange chromatography step was applied to the solution to separate OAg, that is not retained and elutes in the flow-through, from ionic contaminants such as K-Ag, DNA, and residual core. As a final step, ultrafiltration was performed on the purified OAg solution to achieve the target buffer exchange. The process was successfully applied to five different O types, and Table 3 summarizes the characteristics of the final products, confirming the very high levels of purity obtained for all of them.

### 3.3. Purification Methods Developed Applied to the Structural Determination of New K- and O-Loci Saccharide Products

#### 3.3.1. Structural Determination of K-Ag from Strain 4998 (KL107)

The K-Ag from strain 4998 was genomically identified, through the Kaptive tool [34], as a potential new antigen (KL107). The PS was purified, as previously described, after boiling bacteria without OAc, and gave PS with <2% protein and <1% DNA impurities. It showed an average MW of 105 KDa after sonication. HPAEC-PAD analysis indicated the presence of mannose (Man), glucose (Glc), and glucuronic acid (GlcA). Composition analysis using methanolysis, followed by trimethylsilylation derivatisation and gas liquid chromatography–mass spectrometry (GLC-MS) analysis, confirmed the presence of Man, Glc, and GlcA in the molar ratio of 1.0:1.3:1.0. The linkage analysis of the neutral sugars, performed by means of the GLC-MS analysis of the partially methylated alditol acetates, identified 3-Glc*p*, 4-Glc*p*, and 3,4-Man*p* in the molar ratio of 1.2:1.3:1.0. GlcA, not detected by means of methylation analysis, was assumed to be terminal and attached to the 3,4-linked Man. These chemical analyses indicated a tetrasaccharide RU.

**Figure 2:**
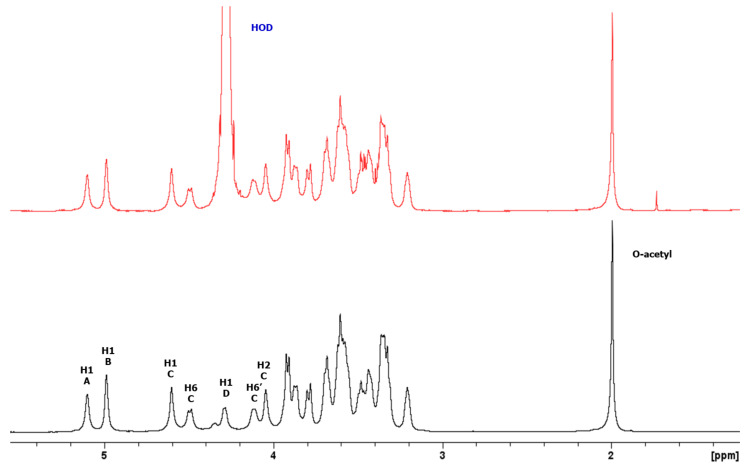
^1^H NMR 1D/1D DOSY overlay of K-Ag from strain 4998 recorded at 600 MHz and 343 K. DOSY removes the signals from low-molecular-weight compounds (HOD and residual solvent) to show signals from the O-acetylated tetrasaccharide RU. Small peaks are from the non-O-acetylated RU.

Detailed 1D and 2D ^1^H and ^13^C NMR experiments were performed on the K-Ag sample. The 1D NMR and DOSY spectra (Figure 2) showed that K-Ag contains four main anomeric signals at 5.28, 5.17, 4.78, and 4.47 ppm (designated A to D) and an O-acetyl peak at 2.17 ppm, consistent with the presence of an O-acetylated tetrasaccharide RU. The two signals at 4.66 and 4.31 ppm were subsequently shown to be deshielded methylene protons (H6 and H6′), characteristic of O-acetylation at C6.

The presence of the O-acetylated tetrasaccharide was corroborated by 1D ^13^C NMR, which gave four anomeric, three methylene group (confirmed by 1D ^13^C-DEPT), and one OAc methyl signals. Two deshielded C=O signals were attributed to C6 of GlcA and the O-acetyl group.

**Figure 3:**
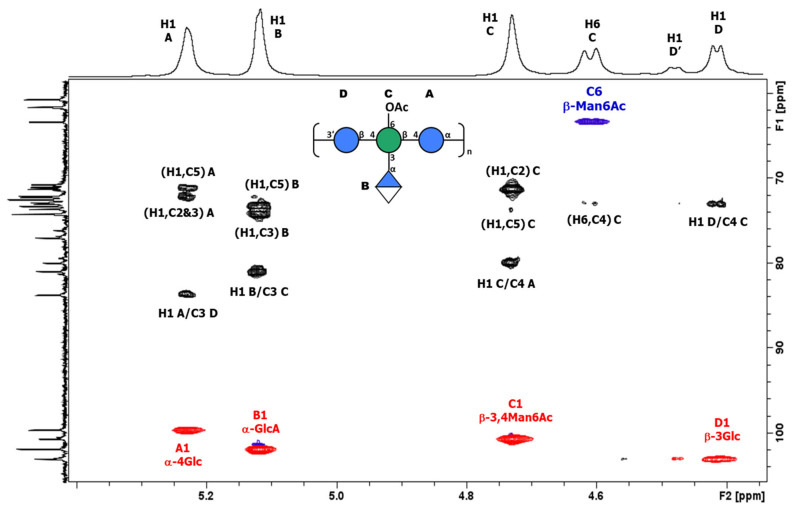
^1^H-^13^C NMR HSQC (red)/HMBC (black) overlay of the anomeric region of K-Ag from strain 4998 recorded at 600 MHz and 343 K. The intra- and inter-residue crosspeaks for the O-acetylated tetrasaccharide RU are labeled (A = α-Glc, B = α-GlcA, C = β-Man6Ac and D = β-Glc).

An overlay of COSY/TOCSY (Figure S4) and a series of 1D TOCSY and NOESY experiments allowed full assignments of the four spin systems and identified the residues as α-Glc (A), α-GlcA (B), β-Man (C), and β-Glc (D), with O-acetylation on C6 of β-Man (C). Small crosspeaks are from the non-O-acetylated RU. All the proton/carbon crosspeaks in the HSQC-DEPT spectrum (Figure S5) were identified from proton assignments aided by HMBC long-range ^1^H/^13^C intra-residue correlations. HMBC gave diagnostic intra-residue correlations from H5 and H4 of residue B to the folded COOH signal, confirming residue B as α-GlcA, and correlations from H6 and H6′ of residue C to the C=O signal of OAc, confirming residue C as β-Man6Ac. The overlay with HSQC/HMBC of the anomeric region (Figure 3) gave the following intra-residue correlations: H1 to C3 and C5 for α-Glc (A) and α-GlcA (B), and H1 to C2 and C5 for β-Man6Ac.

The key inter-residue correlations of H1 of α-Glc (A) to C3 of β-Glc (D), H1 of α-GlcA (B) to C3 of β-Man6Ac (C), H1 of β-Man6Ac (C) to C4 of α-Glc (A), and H1 of β-Glc (D) to C4 of β-Man6Ac (C) establish the linkage positions and sequence of KL107 as: 


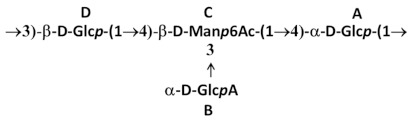


The chemical shift data are collected in Table S2 and are in agreement with the NMR data reported for the non-acetylated tetrasaccharide of the K2 serotype [35], with minor differences due to the O-acetylation of C6 of β-Man6Ac as recently reported [36]. Thus, strain 4998 (KL107) is identified as *Klebsiella pneumoniae* serotype K2 with 86% O-acetylation on C6 of β-Man. Small peaks are present from the non-acetylated RU, the most notable being the small doublet at 4.53 ppm from H1 of β-Glc attached to C4 of β-Man. The NMR assignments established permitted the screening of O-acetylation levels of other K2 strains. Other strains gave different % of O-acetylation (Table S1), confirming that the purification method developed allows the retention of O-Ac groups to be maintained.

#### 3.3.2. Structural Determination of O-Ag from Strain 7008B (OL102)

**Figure 4:**
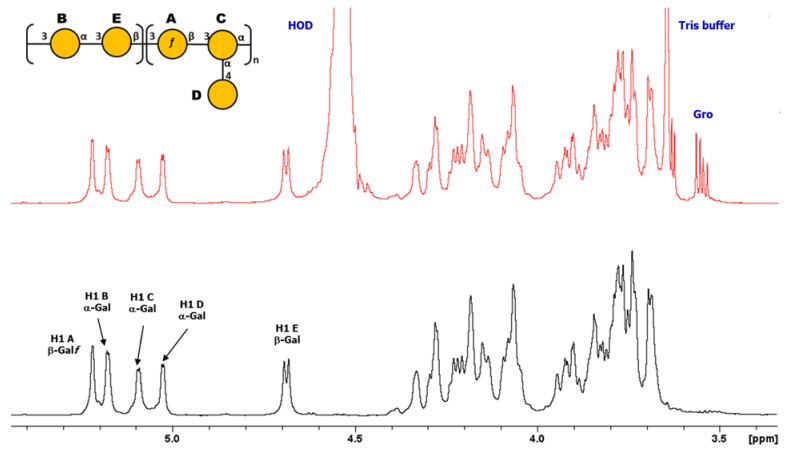
^1^H NMR 1D/1D DOSY overlay of O-Ag from strain 7008B recorded at 600 MHz and 323 K. DOSY removes the signals from low-molecular-weight compounds (HOD, buffer, and glycerol) to show sharp signals from the O-Ag pentasaccharide RU.

The O-Ag from strain 7008B was genomically identified through the Kaptive tool [34] as a potential new antigen (OL102). The O-Ag was purified according to the protocol described and was characterized to contain <4% protein and <1% DNA impurities. It had an average MW of 30 kDa. Sugar composition using both HPAEC-PAD and GLC-MS (after methanolysis and derivatization) showed the presence of galactose only, a common feature of many Kp O-Ags. Detailed 1D and 2D ^1^H and ^13^C NMR experiments were performed on the O-Ag sample. The 1D NMR and DOSY spectra (Figure 4) showed that O-Ag contains five anomeric signals (designated A to E) which were shown to be β-Gal*f*, three aα-Gal*p*, and β-Gal*p* from a pentasaccharide repeating unit (RU), together with residual tris buffer and glycerol (both removed by DOSY).

**Figure 5:**
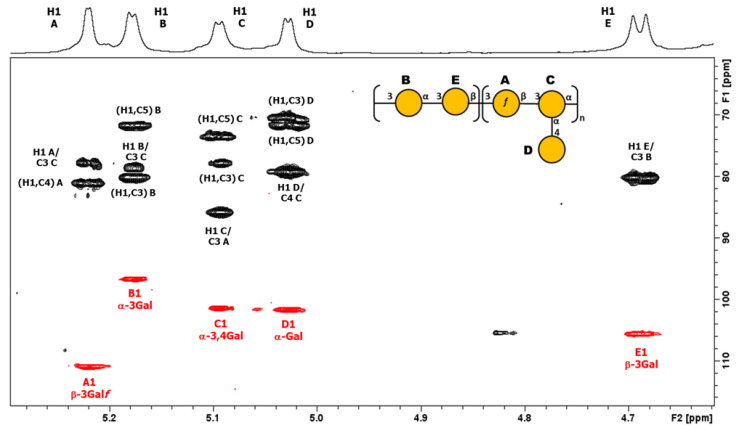
^1^H-^13^C NMR HSQC (red)/HMBC (black) overlay of the anomeric region O-Ag from strain 7008B recorded at 600 MHz and 323 K. The intra- and inter-residue crosspeaks for the KpO1v2 pentasaccharide RU are labeled (A = β-3Gal*f*, B = α-3Gal*p*, C = α-3,4Gal*p*, D= α-Gal*p* and E = β-3Gal*p*).

The proton assignments of most of the pentasaccharide RU were established using DOSY-TOCSY, performed using a mixing time of 180 ms (Figure S6). All the proton/carbon crosspeaks in the HSQC-DEPT spectrum (Figure S7) were identified from proton assignments aided by HMBC long-range ^1^H/^13^C intra-residue correlations. The HSQC/HMBC overlay of the anomeric region (Figure 5) gave the following intra-residue correlations: H1 of β-Gal*f* (A) to C4 and H1 of α-Gal*p* (B, C, and D) to C3 and C5, as expected for a-sugars. The key inter-residue correlations labeled establish the linkage positions and sequence of the O-Ag from strain 7008B (OL102) to be exactly as reported for KpO1v2, with a disaccharide RU (gal-I) and a trisaccharide RU (gal-III) [19].

The gal-I RU was elucidated from H1 of α-Gal*p* (B) to C3 of β-Gal*p* (E) and H1 of βGal*p* (E) to C3 of α-Gal*p* (B), and the gal-III RU from H1 of β-Gal*f* (A) to C3 of α-Gal*p* (C), and H1 of α-Gal*p* (C) to C3 of α-Gal*f* (A), while H1 of terminal α-Gal*p* (D) gave a crosspeak to C4 of α-Gal*p* (C). Thus, the O-Ag produced by strain 7008B, initially identified as a potential new antigen OL102 from the BARNARD study, was classified as having the KpO1v2 [2 + 3] RU. 

## 4. Discussion

In recent years, Kp has gained public health attention as an opportunistic agent, highly resistant to antibiotics, causing a number of severe infections in otherwise healthy individuals [16]. Importantly, Kp is also a leading cause of neonatal sepsis in developing countries. The Child Health and Mortality Prevention Surveillance (CHAMPS) Network, aiming to collect and analyze longitudinal data on under-5 mortality in seven countries in sub-Saharan Africa and South Asia, identified Kp among the most common pathogens causing neonatal deaths [37]. Similar findings came from the Burden of Antibiotic Resistance in Neonates from Developing Societies (BARNARDS) study and the Global Neonatal Sepsis Observational Study (NeoOBS), where the need to recognize Kp as one of the most important neonatal pathogens in LMICs was clearly highlighted [38,39]. Also, it has been estimated that around one million newborns die per year within the first 4 weeks of life because of bloodstream infection with Kp bacteria, and it has been estimated that maternal Kp vaccination could reduce deaths caused by neonatal sepsis in many LMICs by nearly 15% [40]. Many approaches currently in development for a Kp vaccine target K- or O-Ags, which are well-characterized virulence factors exposed on the bacterial surface [7]. One of the major challenges associated with the development of a polysaccharide-based vaccine against Kp is linked to the great diversity of such PSs, that determine serotype specificity, especially in the case of K-Ag, with more than 150 K-loci identified so far. Moreover, the structure of many K-Ags is unknown and no studies have been performed to investigate phenotypic variation among the most prevalent K-serotypes based on regional or temporal differences to support the design of an optimal vaccine.

In this work, a generic purification method for Kp K-Ags was successfully developed and applied to K-serotypes irrespective of their structural variation. This new process allowed the efficient production of K-Ags of high purity, with extremely low percentages of protein and nucleic acid impurities, while also preserving their structural integrity. This is critical when the impact of certain substituents (e.g., O-acetylation) on the immune response of corresponding vaccines need to be investigated [13], and will allow specific implications for Kp vaccine design and efficacy to be explored.

In the literature, very few methods for Kp K-Ag purification have been published and most of them involve historical procedures [30,41,42]. The purification method developed back in 1971 by Gormus et al., applied to K-Ag serotype 6, included the treatment of the sample with toxic reagents such as phenol. In another work, dating back to 1985, Cryz et al. isolated and purified 18 K-Ags, using chloroform and butanol. The use of phenol or chlorinated solvents traditionally used for polysaccharide purification requires the addition of further steps to be eliminated, consequently decreasing the yield and increasing the complexity of the process. More recently, Ovchinnikova et al. published on the purification of K2 by making use of enzymes such as DNAse, RNAse, and proteinase K. Compared to the already-existing processes, our method is based on a fractional precipitation with CTAB and avoids the use of toxic reagents and enzymes. Furthermore, one key advantage of the process relies on its versatility of application, since it was demonstrated to work properly with structurally different K-Ags. When it comes to developing multivalent vaccines, as in the case of Kp, it is essential to have a generic method; otherwise, the development of an ad hoc method for each antigen will be too expensive and time-consuming. 

K-Ags are non-covalently linked to the bacterial membrane and indeed can be released in the supernatant by just boiling the bacteria. Vice versa, O-Ags are attached to the membrane through the lipid A anchor, and acid treatment is needed for their release. Treatment with acetic acid allows the extraction of both K-Ags and O-Ags from only one fermentation broth. The method already established for O-Ag purification [24,29] was slightly modified in this study to allow for the removal of K-Ag chains.

The acetic acid treatment of bacteria resulted in a slight increase in K-Ag yields, but can affect their size. This is not a problem for the synthesis of glycoconjugates, where very high PS molecular mass results in highly viscous products that are difficult to purify and reproduce consistently [43,44]. However, in the case of acid-labile substituents, e.g., pyruvate, acetic acid treatment has to be avoided. In contrast, O-acetyl groups are alkali-labile and not affected by acetic acid treatment.

The purification processes developed here were applied to define the structures of K-Ags and O-Ags from isolates coming from the BARNARDS study that were classified by their locus (KL and OL) and identified as potential new antigens. However, the products from both 4998 and 70008B strains (predicted as KL107 and OL102) [34] turned out to be known structures, O-acetylated K2 and O1v2, respectively, thereby demonstrating how prediction may need chemotyping confirmation for the appropriate classification of Kp serotypes. 

## 5. Conclusions

Herein we have developed and applied a high-throughput method for the purification and analysis of surface carbohydrates from Kp that will be extended to a larger collection of serotypes from the BARNARDS study to confirm genotypic information and verify PS structures and features. Findings will facilitate the understanding of their variability and investigation of potential cross-reactivity among serotypes. Overall, this work will support the design of a multivalent vaccine against *Klebsiella pneumoniae* infections.

## Figures and Tables

**Table 1:** K-Ag purification: characterization of intermediates.

Purification Intermediates	Sugar Step Yield %				Protein %				DNA %			
	K2	K15	K64	K64^†^	K2	K15	K64	K64 ^†^	K2	K15	K64	K64 ^†^
Step 1: Supernatant after DNA precipitation with CTAB	87 *	75 *	103 *	89 *	12.2	9.3	194	48.3	82.0	7.7	10.6	12.1
Step 2: Pellet solubilized in CaCl_2_/NaCl ^#^	88	75	62	92	6.8	7.1	11.8	5.0	62.5	5.6	1.8	3.0
Step 3: Pellet post EtOH precipitation	80	56	94	58	5.5	5.8	2.8	3.8	3.6	1.8	1.1	5.1
Step 4: Final product post 10 K and 0.22 μm filtration	71	96	107	102	2.0	1.6	0.6	1.1	0.4	0.7	0.1	0.7

* Calculated with respect to the K-Ag amount quantified in the supernatant soon after extraction from bacteria. ^#^ Note that this intermediate was characterized after treatment by means of dilution and following PS precipitation. The characterization of K64 purification was performed on PSs extracted by boiling both with (K64) and without AcOH (K64 ^†^).

**Table 2:** Impact of AcOH on the saccharide yield, size, and level of O-acetylation.

KAg Type	Extraction Method	Amount Obtained *	Molecular Weight	OAc Level %
K2	H_2_O	29 mg	530 kDa	~55%
	AcOH	36 mg	309 kDa	~45%
K62	H_2_O	68 mg	357 kDa	na
	AcOH	74 mg	160 kDa	na
K64	H_2_O	11 mg	1390 kDa	~65%
	AcOH	12 mg	391 kDa	~63%
K15	AcOH	50 mg	58 kDa	~97%

* refers to the amount obtained, starting from 10 plates for K2, K15, and K62, and 2 plates for K64.

**Table 3:** K- and O-Ag purified from different strains.

Strain	K-Locus	Structure		K-Ag Purity %
NCTC9135	K15	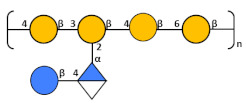	[32]	98%
NCTC9145	K25	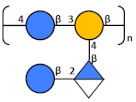	[33]	99%
12641B	K102	Unknown		99%
17648	K105			99%
27870B	K108			97%
19679	K112			99%
24745-2	K116			97%
10405	K117			98%
773	K122			97%
30405	K128			98%
34473	K136			97%
**Strain**	**O-locus**	**Structure**		**O-Ag Purity %**
7008B	O1v2	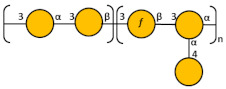		99%
1217	O3a	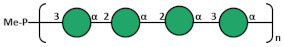	[20]	99%
24649	O3b	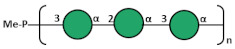	[20]	99%
25827	O4	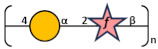	[21]	98%
29867	O5	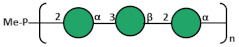	[18]	98%

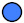
 Glucose (Glc); 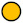 Galactose (Gal); 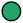 Mannose (Man); 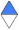 Glucuronic Acid (GlcA); 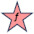 Ribose (Rib*f*).

## Data Availability

The authors declare that data are contained within the article and in the Supplementary Materials.

## Supplementary Materials

The following supporting information can be downloaded at: https://www.mdpi.com/article/10.3390/biology13040256/s1.
