# Supplementary material for: Development and Application of a High-Throughput Method for the Purification and Analysis of Surface Carbohydrates from Klebsiella pneumoniae
Source: Biology (Basel). 2024 Apr 12;13(4):256. doi: 10.3390/biology13040256 (PMC11048683; doi:10.3390/biology13040256)
Supplement: Supplementary file 1 [file biology-13-00256-s001.zip › biology-2929665-supplementary.pdf]

Supplementary Information

Development and application of a high-throughput method for the purification and analysis of surface carbohydrates from *Klebsiella pneumoniae*

Francesca Nonne[1], Mariagrazia Molfetta[1], Rebecca Nappini[1,2], Chiara La Guidara[1], Roberta Di Benedetto[1], Siwaphiwe Mfana[3], Barbara Bellich[4*], Maria Michelina Raso[1], Gianmarco Gasperini[5], Renzo Alfini[1], Paola Cescutti[2], Francesco Berlanda Scorza[1], Neil Ravenscroft[3], Francesca Micoli[1], Carlo Giannelli[1]

[1]GSK Vaccines Institute for Global Health, Siena, Italy

[2] Department of Life Science, University of Trieste, Trieste, Italy

[3] Department of Chemistry, University of Cape Town, South Africa.

[4] Department of Advanced Translational Diagnostics, Institute for Maternal and Child Health, IRCCS "Burlo Garofolo", Trieste, Italy

[5] GSK, Siena, Italy

* When the experiments were performed BB was working at the Department of Life Sciences, University of Trieste, Trieste, Italy

Table S1. Evaluation of OAc level after KAg purification

KAg type	Strain	Extraction method	OAc %
K2	NCTC13810	H_2O	~55%
	NCTC13810	AcOH	~45%
	NCTC11228	AcOH	~98%
	5765B	H_2O	~77%

Table S2: Complete ^{1}H and ^{13}C NMR chemical shifts data of the sugar residues in the polysaccharide produced by strain 4998 (identified as O-acetylated K2) with glycosylation shifts shown in brackets below the carbon chemical shifts; the linkage carbons are underlined.

Residue	H1<br>C1	H2<br>C2	H3<br>C3	H4<br>C4	H5<br>C5	H6<br>C6	OAc
**A**<br>**→4)-α-D-Glc*p*-(1→**	5.28<br>99.79<br>(6.80)	3.61<br>72.27<br>(-0.20)	3.86<br>72.39<br>(-1.39)	3.66<br><u>80.13</u><br>(9.42)	4.05<br>71.21<br>(-1.16)	3.75; 3.79<br>60.87<br>(-0.97)	-
**B**<br>**α-D-Glc*p*A-(1→**	5.17<br>102.04<br>(9.08)	3.53<br>72.65<br>(0.39)	3.79<br>74.40<br>(0.88)	3.51<br>72.72<br>(-0.19)	4.10<br>73.45<br>(0.98)	-<br>176.89<br>(-0.53)	-
**C**<br>**→3,4)-β-D-Man*p*6Ac-(1→**	4.78<br>100.86<br>(6.31)	4.23<br>71.42<br>(-0.71)	3.86<br><u>81.14</u><br>(7.11)	4.12<br><u>73.21</u><br>(5.52)	3.80<br>73.79<br>(-3.21)	4.66; 4.31<br>63.51<br>(1.52)	2.17<br>21.13<br>174.61
**D**<br>**→3)-β-D-Glc*p*-(1→**	4.47<br>103.20<br>(6.36)	3.39<br>73.03<br>(-2.17)	3.63<br><u>83.92</u><br>(7.16)	3.56<br>70.91<br>(0.20)	3.50<br>77.18<br>(0.42)	3.75; 3.97<br>61.76<br>(-0.08)	-

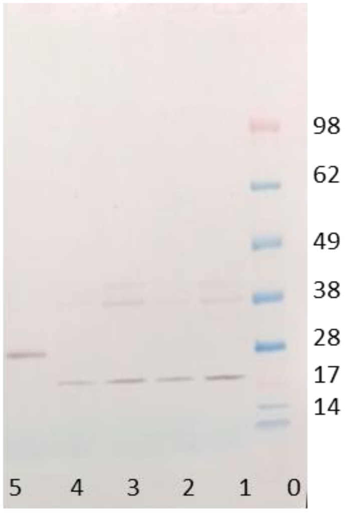

0: Marker
1: K62 extracted and purified from LB plate
2: K62 extracted and purified from WF plate
3: K64 extracted and purified from LB plate
4: K64 extracted and purified from WF plate
5: Recombinant form of MrkA – MW= 23KDa vs 20kDa of native MrkA monomer

Figure S1. Western Blot analyses shows recognition of K62 and K64 samples by an anti-MrkA antibody confirming the presence of MrkA protein in the purified KAgs.

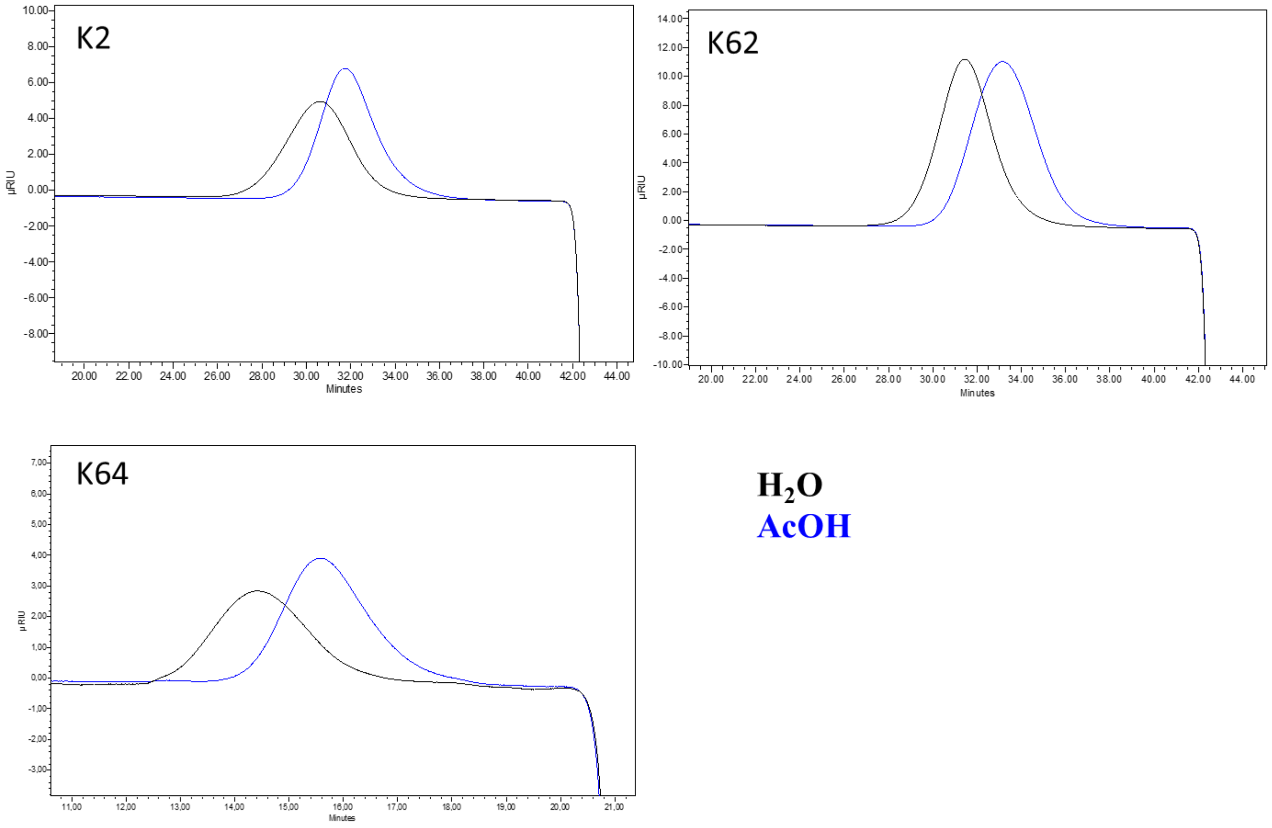

Figure S2. HPLC-SEC profiles highlight differences in the size of purified K-Ag obtained from bacterial extraction with and without AcOH.

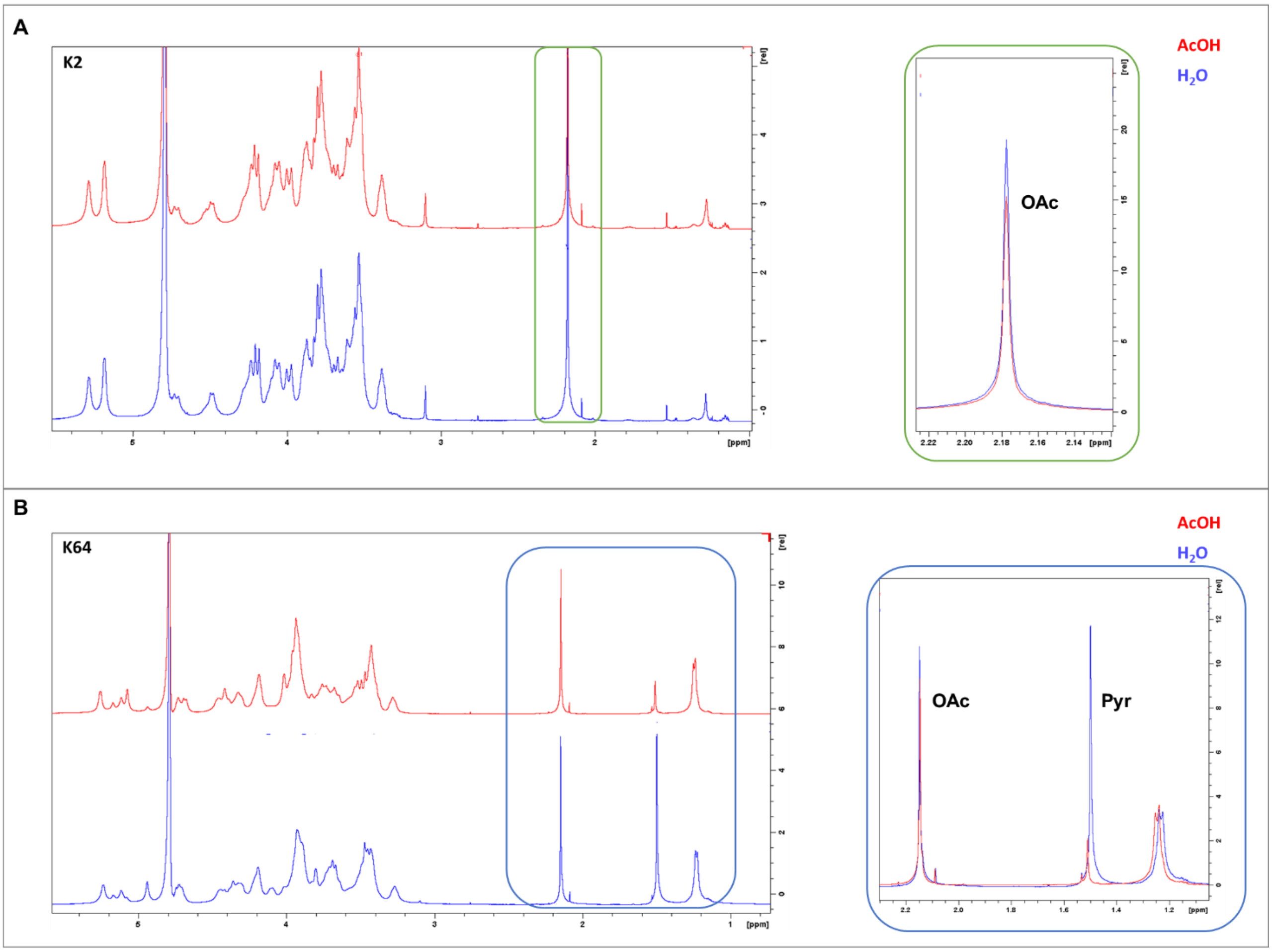

Figure S3. Impact of AcOH on K2 (A) and K64 (B) PS structure integrity by NMR analysis

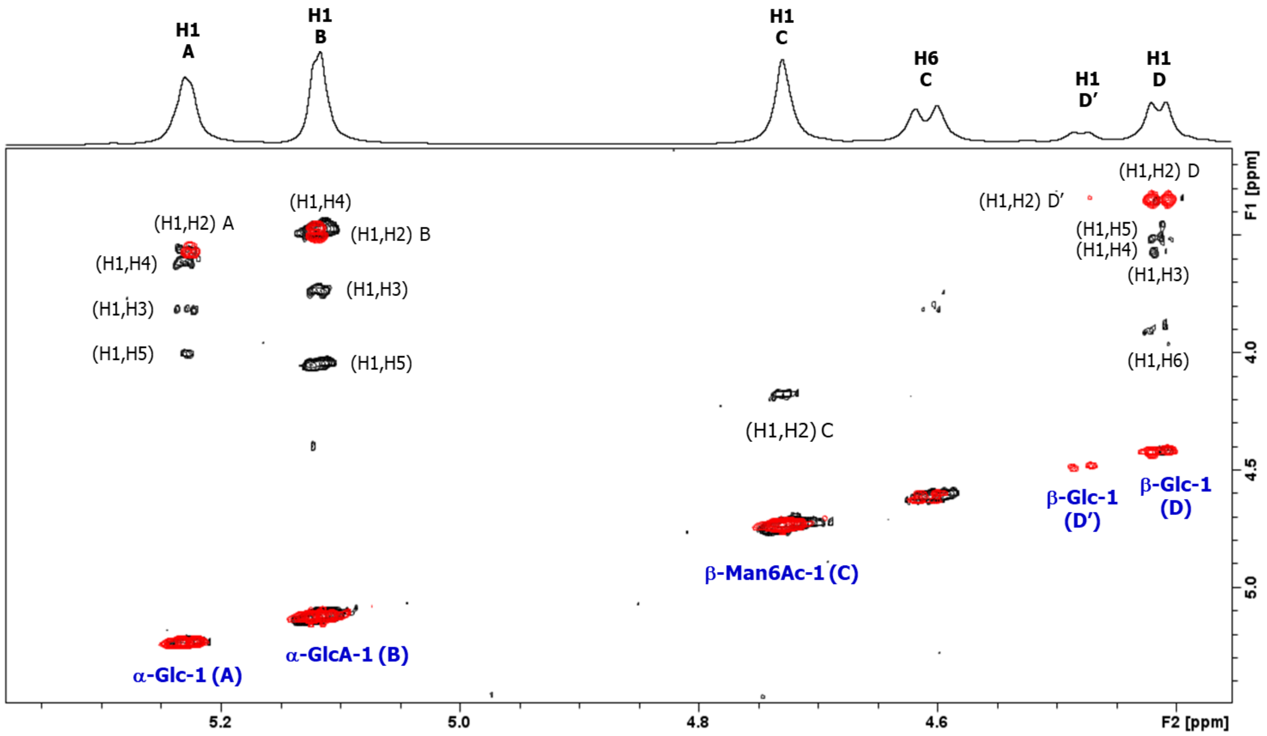

Figure S4. ^{1}H-^{1}H NMR COSY (red)/TOCSY (black) overlay of the anomeric region of K-Ag from strain 4998 recorded at 600 MHz and 343 K. The crosspeaks for the O-acetylated tetrasaccharide RU are labeled (A = α-Glc, B = α-GlcA, C = β-Man6Ac and D = β-Glc).

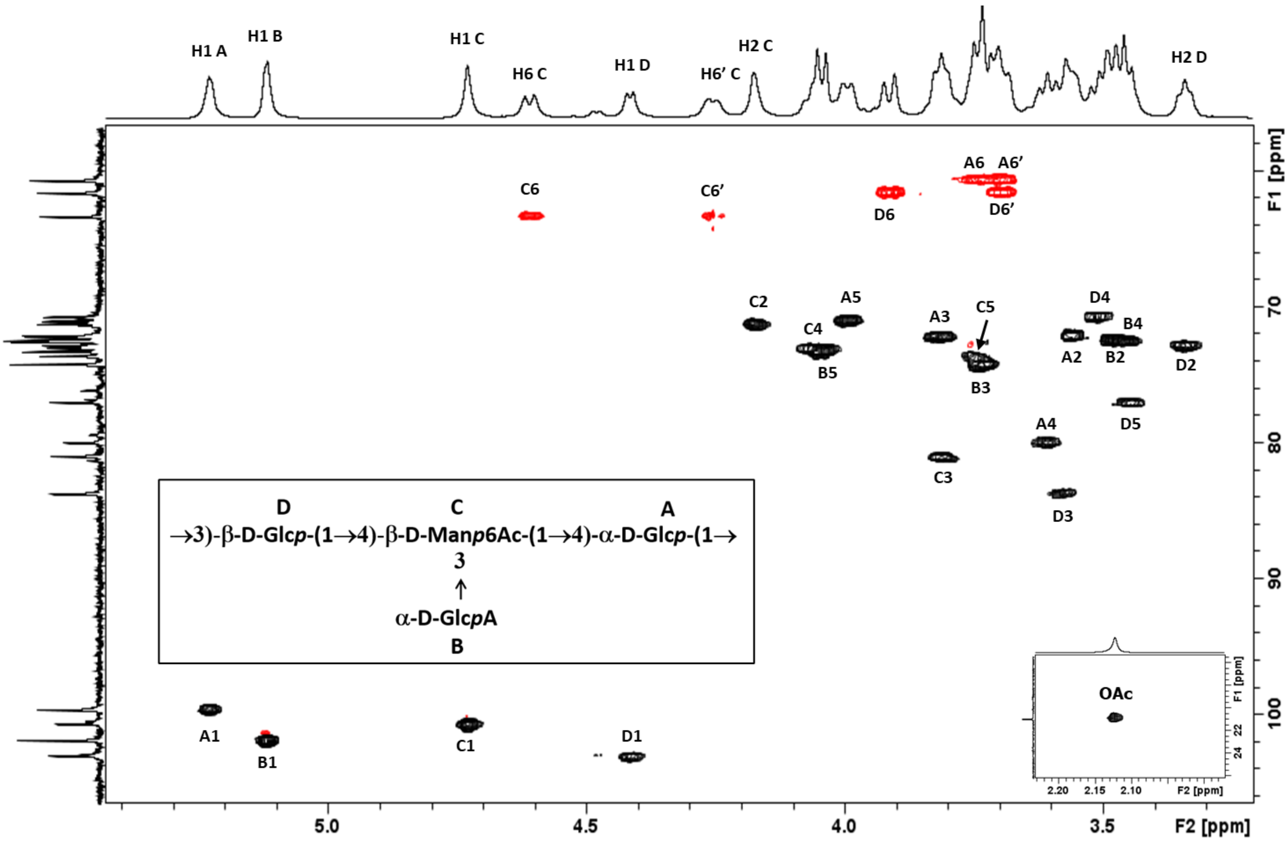

Figure S5. The HSQC-DEPT spectrum of K-Ag from strain 4998 recorded at 600 MHz and 343 K, methylene signals are inverted (in red), the inset shows the methyl region. All the O-acetylated tetrasaccharide repeat unit proton/carbon crosspeaks have been labeled according to the carbon atom of the corresponding residue (A = α-Glc, B = α-GlcA, C = β-Man6Ac and D = β-Glc).

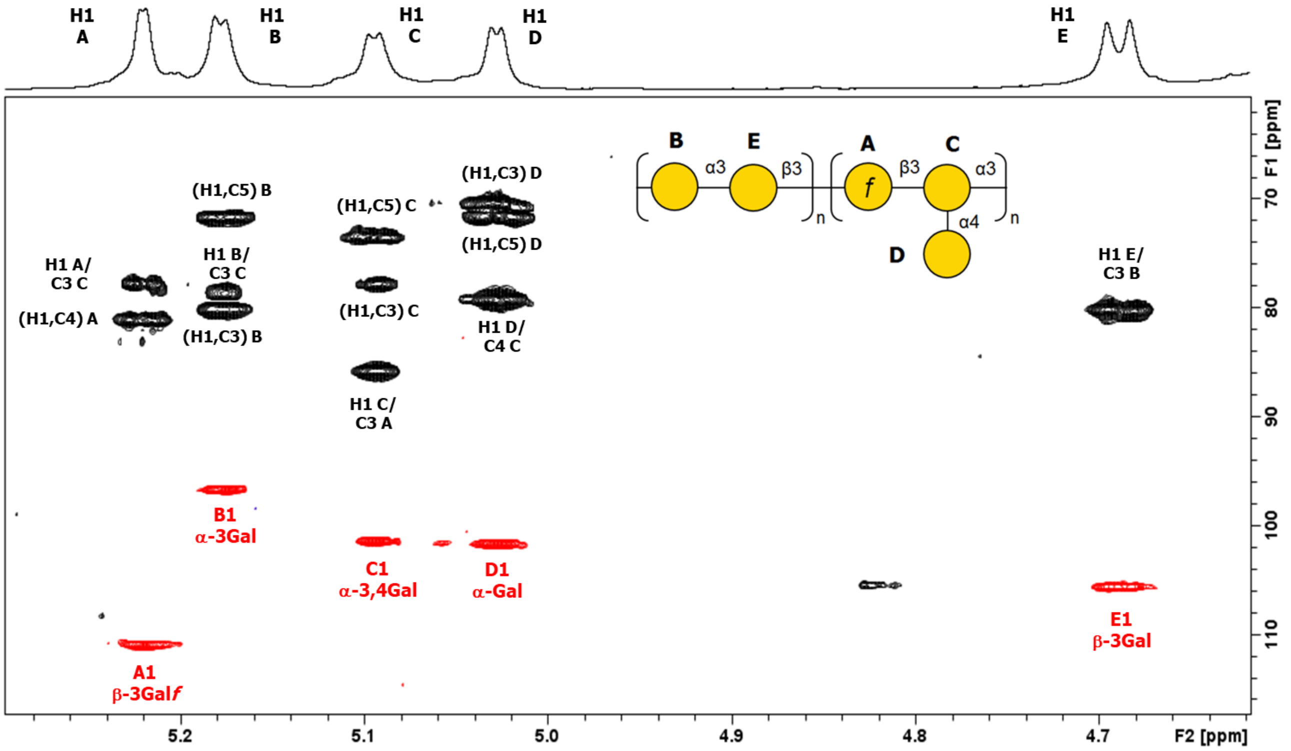

Figure S6. ^{1}H-^{1}H NMR COSY (red)/TOCSY (black) overlay of the anomeric region of O-Ag from strain 7008B recorded at 600 MHz and 323 K. The crosspeaks for the pentasaccharide repeat unit have been labeled (A = β-3Gal*f*, B = α-3Gal*p*, C = α-3,4Gal*p*, D= α-Gal*p* and E = β-3Gal*p*).

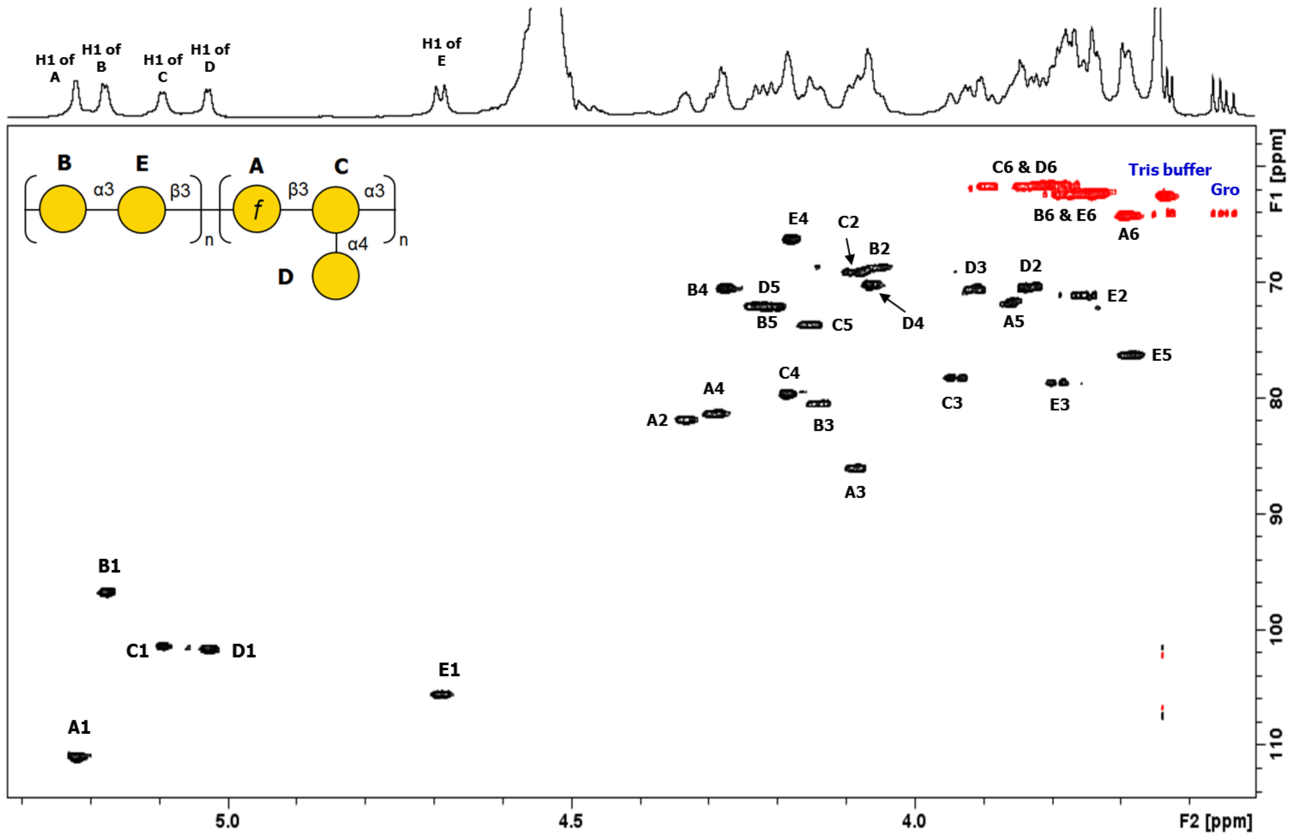

Figure S7. The HSQC-DEPT spectrum of O-Ag from strain 7008B recorded at 600 MHz and 323 K, methylene signals are inverted (in red). All the pentasaccharide repeat unit proton/carbon crosspeaks have been labeled according to the carbon atom of the corresponding residue in the RU (A = β-3Gal*f*, B = α-3Gal*p*, C = α-3,4Gal*p*, D= α-Gal*p* and E = β-3Gal*p*). Additional peaks are due to buffer and glycerol (Gro).
